# Utilizing microsatellite instability and immunohistochemistry to clinically interpret a novel germline mismatch repair mutation of uncertain significance

**DOI:** 10.1186/1897-4287-9-S1-P9

**Published:** 2011-03-10

**Authors:** Whitney L Ducaine, Lindsay Dohany, Richard Zekman, Dana Zakalik

**Affiliations:** 1 Cancer Genetics Program, William Beaumont Hospital, Royal Oak, Michigan, USA

## Background

Lynch syndrome (LS) is responsible for around 2-3% of all colorectal cancers[1]. Germline mutations within one of four mismatch repair (MMR) genes, *MLH1, MSH2, MSH6,* or *PMS2*, cause an increased risk in colorectal, endometrial, urinary tract, and other cancers. Tumor screening, by microsatellite instability (MSI) and immunohistochemistry (IHC) analysis, can be utilized prior to mutation analysis in order to streamline the testing process[2]. The following describes the use of MSI and IHC to aid in the clinical interpretation of a novel MMR mutation of uncertain significance and the direct impact on cancer detection.

## Materials and methods/results

Family X presented to our cancer genetics clinic with a previously identified *MSH2* MMR gene variant of uncertain significance, 1227del12, detected in Sib 1, who was diagnosed with synchronous colon cancers at age 53. This mutation is an in-frame deletion of four amino acids (Gln, Gly, Ile, Asn) in exon 7, which is located in an alpha-helix turn of the protein. Sib 2 had a bladder cancer at 56 years, and Sib 3 had a ureter cancer at 62 years. Sibs 2 and 3 were tested by standard DNA sequencing for the specific VUS[6]. All three Sibs carry the *MSH2* 1227del12 mutation. This mutation has not been previously reported in the literature; hence clinical interpretation of this VUS was limited. For this reason, coordination of MSI and IHC was pursued for the three Sibs’ tumors. MSI was high for Sibs 2 and 3 and unable to be performed on Sib 1. All three Sibs’ tumors showed concurrent loss of MSH2 and MSH6 by IHC.

## Conclusions

This IHC pattern is suspicious of an underlying germline mutation within the *MSH2* gene[4,5]. In addition, both of the tested tumors were MSI-high. Given these results, and a significant family history, Family X was clinically diagnosed with LS, with the *MSH2* VUS as the likely cause. Relatives with the VUS were informed to follow LS management recommendations, and those without the VUS should undergo high risk screening[2,3]. Sib 2 then underwent a colonoscopy one year from a previous unremarkable colonoscopy, and a Stage I, 1.2 cm proximal colon adenocarcinoma with focal mucin production was detected.

This case illustrates the benefit of tumor screening and genetic professional expertise in the clinical interpretation of a MMR VUS as it applies to clinical diagnosis, early detection and management of high risk families.
